# Insulin directly stimulates mitochondrial glucose oxidation in the heart

**DOI:** 10.1186/s12933-020-01177-3

**Published:** 2020-12-07

**Authors:** Qutuba G. Karwi, Cory S. Wagg, Tariq R. Altamimi, Golam M. Uddin, Kim L. Ho, Ahmed M. Darwesh, John M. Seubert, Gary D. Lopaschuk

**Affiliations:** 1 Cardiovascular Research Centre, University of Alberta, 423 Heritage Medical Research Centre, Edmonton, AB T6G 2S2 Canada; 2 Mazankowski Alberta Heart Institute, University of Alberta, Edmonton, AB Canada; 3 Faculty of Pharmacy and Pharmaceutical Sciences, University of Alberta, Edmonton, AB Canada; 4 Department of Pharmacology, College of Medicine, University of Diyala, Diyala, Iraq

**Keywords:** Glucose oxidation, Insulin signaling, Protein kinase B (akt), Protein kinase C-delta (PKC-δ), Glycogen synthase kinase-3 beta (GSK-3β), Mitochondria

## Abstract

**Background:**

Glucose oxidation is a major contributor to myocardial energy production and its contribution is orchestrated by insulin. While insulin can increase glucose oxidation indirectly by enhancing glucose uptake and glycolysis, it also directly stimulates mitochondrial glucose oxidation, independent of increasing glucose uptake or glycolysis, through activating mitochondrial pyruvate dehydrogenase (PDH), the rate-limiting enzyme of glucose oxidation. However, how insulin directly stimulates PDH is not known. To determine this, we characterized the impacts of modifying mitochondrial insulin signaling kinases, namely protein kinase B (Akt), protein kinase C-delta (PKC-δ) and glycogen synthase kinase-3 beta (GSK-3β), on the direct insulin stimulation of glucose oxidation.

**Methods:**

We employed an isolated working mouse heart model to measure the effect of insulin on cardiac glycolysis, glucose oxidation and fatty acid oxidation and how that could be affected when mitochondrial Akt, PKC-δ or GSK-3β is disturbed using pharmacological modulators. We also used differential centrifugation to isolate mitochondrial and cytosol fraction to examine the activity of Akt, PKC-δ and GSK-3β between these fractions. Data were analyzed using unpaired t-test and two-way ANOVA.

**Results:**

Here we show that insulin-stimulated phosphorylation of mitochondrial Akt is a prerequisite for transducing insulin’s direct stimulation of glucose oxidation. Inhibition of mitochondrial Akt completely abolishes insulin-stimulated glucose oxidation, independent of glucose uptake or glycolysis. We also show a novel role of mitochondrial PKC-δ in modulating mitochondrial glucose oxidation. Inhibition of mitochondrial PKC-δ mimics insulin stimulation of glucose oxidation and mitochondrial Akt. We also demonstrate that inhibition of mitochondrial GSK3β phosphorylation does not influence insulin-stimulated glucose oxidation.

**Conclusion:**

We identify, for the first time, insulin-stimulated mitochondrial Akt as a prerequisite transmitter of the insulin signal that directly stimulates cardiac glucose oxidation. These novel findings suggest that targeting mitochondrial Akt is a potential therapeutic approach to enhance cardiac insulin sensitivity in condition such as heart failure, diabetes and obesity.

## Background

The mitochondrial oxidation of pyruvate derived from glucose (glucose oxidation) is a major source of acetyl CoA for the tricarboxylic acid (TCA) cycle and reducing equivalents for adenosine triphosphate (ATP) production in the heart. Insulin plays a crucial role in cardiac energy metabolism by orchestrating the contribution of oxidative substrates, including glucose and fatty acids, to cardiac ATP production. Insulin causes a switch in cardiac energy substrate preference, by stimulating glucose oxidation and inhibiting fatty acid oxidation [1–6]. Insulin indirectly stimulates glucose oxidation via increasing glucose uptake and subsequent glycolysis that increases pyruvate supply for mitochondrial glucose oxidation by the pyruvate dehydrogenase (PDH) complex, the rate-limiting enzyme of glucose oxidation. In the heart, glucose uptake is mainly mediated by the role glucose transporter 1 and 4 (GLUT1 and GLUT4), which behave differently following insulin stimulation [7]. GLUT4 (the insulin sensitive glucose transporter) translocates from the endosome stores to the sarcolemmal membrane for glucose assimilation following insulin stimulation, while GLUT1 is more localized in the cytosol and less dominant in the sarcolemma [8–10]. GLUT1 is less responsive to insulin but can be activated by 5′AMP-activated protein kinase (AMPK) [8]. Increased glucose uptake drives glycolysis to convert glucose to pyruvate. Pyruvate is then taken up by the mitochondria through the mitochondrial pyruvate carrier (MPC) to be oxidized by PDH. The activity of PDH is largely regulated by its phosphorylation status and it is active when it is dephosphorylated. The PDH complex can be phosphorylated and inhibited by pyruvate dehydrogenase kinase (PDK), while it can be dephosphorylated and activated by pyruvate dehydrogenase phosphatase (PDP).

It has also been shown that insulin can directly stimulate glucose oxidation independent of enhancing glucose uptake or glycolysis [11–14]. The hallmark of this direct stimulation of glucose oxidation by insulin is the dephosphorylation and activation of the mitochondrial PDH complex [11–14]. However, the exact mechanism through which the insulin signal is transmitted from the cell membrane to the mitochondria to activate the PDH complex has not been previously elucidated. The mouse heart has high glycolytic rates that are almost maximized even in the absence of insulin [15, 16]. Because of this, insulin does not have dramatic effects on glucose uptake and glycolysis in the mouse heart [15, 16]. Despite this, insulin still directly stimulates cardiac glucose oxidation, independent of any change in glucose uptake or glycolysis rates [3–5, 15, 16]. How insulin directly stimulates glucose oxidation in the heart is presently not known. However, because of the high insulin-independent glycolytic rates, the mouse heart provides a valuable tool to examine how insulin stimulates glucose oxidation in the heart independent of pyruvate supply to the mitochondria.

Protein kinase B (Akt), glycogen synthase kinase-3 beta (GSK-3β) and protein kinase C-delta (PKC-δ) are key components of the insulin signaling pathway. Interestingly, it has been shown that these kinases can be translocated to the mitochondria following insulin stimulation [17–21]. The translocation of these kinases has also been linked to modulating mitochondrial oxidative phosphorylation. For example, it has been reported that Akt can rapidly be translocated to the mitochondria following insulin stimulation in a PI3K-dependent manner in SH-SY5Y human neuroblastoma cells [17]. Moreover, insulin-stimulated mitochondrial translocation of GSK-3β has also been shown to be associated with inhibition of the PDH complex in primary rat hippocampal cells [18]. Furthermore, mitochondrial translocation of PKC-δ has been linked to the activation of the PDH complex in Hep cell clones and that pharmacological inhibition of PKC-δ translocation abrogates insulin-stimulated glucose oxidation [19]. However, mitochondrial translocation of PKC-δ has also been shown to inhibit the PDH complex activity via activating PDK in fibroblasts [20]. Taken together, it seems plausible to propose that these kinases are potential candidates to transduce insulin signal from the cell membrane to the mitochondria to directly stimulate the PDH complex and glucose oxidation in the heart. Therefore, we hypothesized that stimulating mitochondrial Akt, GSK-3β or PKC-δ is a prerequisite to mediate the direct stimulatory effects of insulin on the PDH complex and cardiac glucose oxidation. We also hypothesized that manipulating the activity of these kinases in the mitochondria will influence the direct stimulatory effect of insulin on glucose oxidation independent of any change in glucose uptake or glycolysis rates.

## Materials and methods

### Animals

All procedures were approved by the University of Alberta Health Sciences Animal Policy and Welfare Committee and the care of mice, and conformed to the guidelines of the Canadian Council on Animal Care. Male and female C57BL/6N mice were sourced from Charles River Laboratories (Wilmington, MA, USA). Mice were housed at the University of Alberta Health Sciences Lab Animal Services facility in a temperature- and humidity-controlled room with a 12 h light dark cycle. The investigation conforms to the *Guide for the Care and Use of Laboratory Animals* published by the US National Institute of Health (NIH Publication No. 85-23, revised 1985).

### Measurement of myocardial energy metabolism

**Fig. 1:**
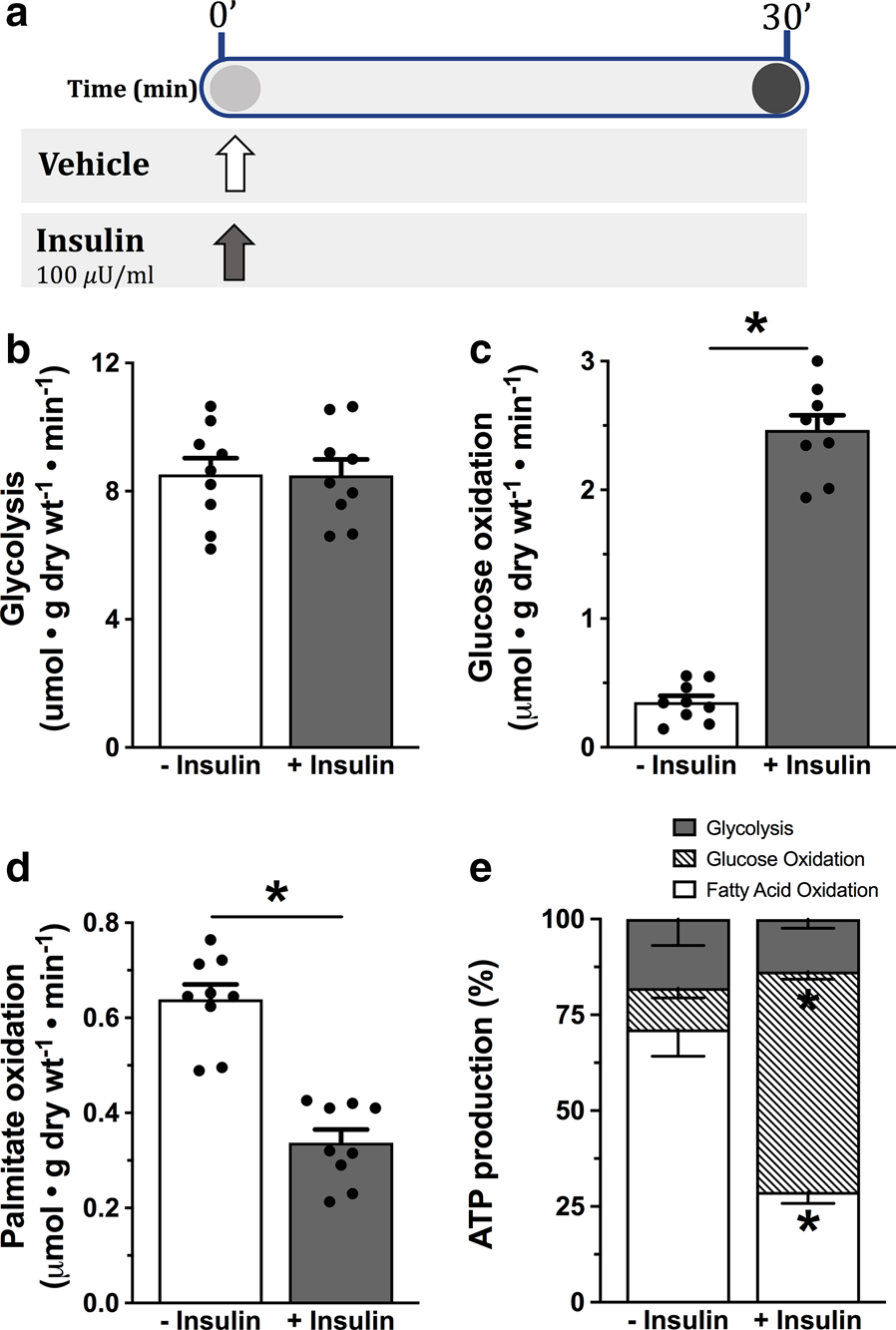
Insulin stimulation of glucose oxidation rates is independent of glycolysis in the mouse heart. **a** Schematic of study design for Study 1. Hearts were perfused in an isolated working heart mode for 30 min with either vehicle or insulin throughout the perfusion protocol. The metabolic profile of the heart was characterized by measuring **b** glycolysis, **c** glucose oxidation and **d** palmitate (fatty acid) oxidation along with **e** their contribution to cardiac ATP production (n = 9 for each experimental group). Arrows indicate the time of adding the vehicle of insulin to the perfusate. Individual values for each group are presented as a scattered plot along with its mean ± SEM. Data were analyzed using an unpaired student t-test. *p < 0.05 compared to the vehicle-treated hearts

This study used isolated working mouse hearts. The mouse heart has high glycolytic rates that are maximally stimulated in the absence of insulin. Therefore, this allowed us to manipulate the activity of Akt, PKC-δ and GSK-3β without having a significant effect on glucose uptake or glycolysis that could potentially influence glucose oxidation. These isolated working hearts also allow us to directly measure basal and insulin-stimulated glucose oxidation rates in the presence and absence of the pharmacological modulators of Akt, PKC-δ and GSK-3β. In Study 1, male and female mice hearts were randomized to be perfused for 30 min with or without insulin (Fig. 1a). Insulin (100 µU/ml) or vehicle (0.03% DMSO) were added at the beginning of the perfusion protocol (n = 9 per group). In Study 2 (Fig. 3a), male and female mice hearts were randomized to be perfused either with vehicle (0.03% DMSO) or one of the pharmacological modulators of insulin signaling kinases, namely LY294002 (PI3K/Akt inhibitor, 10 µM, Millipore Sigma) [22], Akti VIII (Akt inhibitor, 1 µM, Santa Cruz Biotechnology) [23], Bisindolylmaleimide (PKC-δ inhibitor, 1 µM, Santa Cruz Biotechnology) [24], or 3F8 (GSK-3β inhibitor, 5 µM, Santa Cruz Biotechnology) [25] (n = 9 per group). Hearts were perfused for 30 min in the absence of insulin, following which insulin (100 µU/ml) was then added to the perfusate and the hearts were perfused for additional 30 min. In both cohorts, hearts were perfused with Krebs–Henseleit solution (118.5 mM NaCl, 25 mM NaHCO_3_, 4.7 mM KCl, 1.2 mM MgSO_4_, 1.2 mM KH_2_PO_4_, 2.5 mM CaCl_2_) containing 5 mM glucose and 0.8 mM palmitate pre-bound to 3% albumin. The aorta was cannulated with an 18-gauge plastic cannula, while the left atrium was connected to the preload reservoir (oxygenator) by cannulating the pulmonary vein with a 16-gauge steel cannula. The preload line (and perfusate reservoir) was wrapped with a water jacket and heated to 38.5 °C, resulting in a myocardial temperature very close to 37 °C (36.4–36.7 °C) when the heart was operating in the working mode. When the heart was switched from the Langendorff to the working mode, the left atrium was perfused at a preload pressure of 15 mmHg; the left ventricle worked against a hydrostatic column set at a height equivalent to a pressure of 50 mmHg.

Glycolysis, glucose oxidation and palmitate oxidation rates were measured by simultaneously sampling ^14^CO_2_ and ^3^H_2_O produced from the metabolism of [5-^3^H]glucose, [U-^14^C] glucose and [9,10-^3^H] palmitate [4, 16]. For ATP production, the rates of glycolysis, glucose oxidation and fatty acid oxidation were multiplied by the number of ATP molecules produced from each process (i.e. 2, 29 and 105 ATP, respectively). Then, the results were converted into percentages (out of 100%) to compare the contribution of each of these processes to the total cardiac ATP production. To directly measure cardiac oxygen consumption rates, we cannulated the pulmonary artery and oxygen concentrations difference in the buffer were measured between the left atria and the pulmonary artery using in-line oxygen probes (Microelectrodes, USA). The effluent was sampled in triplicates every 10 min during the perfusion protocol and these samples were processed then analyzed and the values for each time point were averaged. To calculate cardiac efficiency throughout the perfusion protocol, we divided the cardiac work by the oxygen consumption at each time point. This approach allows us to correct for any changes in oxygen consumption that are secondary to changes in cardiac function and not due to alterations in cardiac energy metabolism.

### Mitochondrial isolation

At the end of the perfusions the hearts were homogenized using a modified protocol described previously [22]. Briefly, all the procedures were conducted at 4 °C to maintain the integrity of mitochondrial proteins. The hearts were immersed in the homogenization buffer (in 50 ml: 10 mM Tris HCl, 5 tablets of protease inhibitor (Pierce Protease and Phosphatase Inhibitor Mini Tablets, ThermoFisher, catalog A32959), and 250 mmol/l sucrose pH 7.0). The hearts were then chopped into small pieces with small scissors and homogenized with a teflon pestle in a glass tube. Tissue homogenate was centrifuged at 800×*g* for 10 min to pull down tissue debris while the supernatant was collected and centrifuged at 10,000×*g* for 20 min to separate the mitochondrial fraction as a pellet. The supernatant from the second centrifugation was further centrifuged at 105,000×*g* for 60 min to separate microsomes from the cytosolic fraction. For subsequent investigations, mitochondrial and cytosolic fractions were used. Protein concentration in each fraction was measured using Bradford assay (BioRad, USA).

### Immunoblotting analysis

Mitochondrial and cytosolic fractions (30 µg/well) were loaded onto 10% SDS-PAGE to be separated based on molecular weight, then transferred onto nitrocellulose membrane using wet-transfer method, as previously reported [3, 4]. The membrane was then probed with one of the following primary antibodies (1:1000): phospho-Akt Ser^473^ (Cell Signaling, catalog 9271S), Akt (Cell Signaling, catalog 9272S), phospho-GSK-3β Ser^9^ (Cell Signaling, catalog 9322S), GSK-3β (Cell Signaling, catalog 9321S), phospho-PKC-δ Tyr^311^ (Santa Cruz Biotechnology, catalog SC-377560), PKC-δ (Santa Cruz Biotechnology, catalog SC-8402), phospho-PDH-E1α Ser^293^ (Millipore Sigma, catalog ABS204), PDH (Cell Signaling, catalog 3205S). The probed membrane was incubated with the correspondent secondary antibody (1:5000) for 1 h then protein bands were visualized using the Amersham enhanced chemiluminescence kit (Cell Signaling Technologies, Danvers, Massachusetts, USA). Protein bands intensity analysis was performed in a blind fashion using ImageJ program (1.48v, National Institutes of Health USA). Prohibitin (Fitzgerald, catalog 70R-5543) and α-tubulin (Millipore Sigma, catalog T9026) was used as an internal control (loading control) for the mitochondrial and cytosolic fractions, respectively. To account for any possible variation in the band’s intensity due to different exposure times for different membranes, the average for all the samples in the same group was calculated, and each sample was then divided by the average value.

### Statistical analysis

Data passed the Kolmogorov–Smirnov normality test of distribution and are represented as mean ± SEM. An unpaired student t-test was used for comparison between two experimental groups and one-way or two-way analysis of variance (ANOVA) followed by Bonferroni post-hoc test was used for multiple comparison. p < 0.05 was deemed significant.

## Results

### Direct insulin stimulation of glucose oxidation is associated with enhanced phosphorylation of mitochondrial Akt, GSK-3β and PKC-δ

We first aimed to characterize whether direct insulin stimulation of the PDH complex and glucose oxidation is associated with activation of mitochondrial Akt, GSK-3β or PKC-δ in the mouse heart. We randomized C57BL/6N mice hearts to be perfused in an isolated working heart mode either with or without insulin (Fig. 1a). There was also no significant difference in cardiac glycolytic rates in the presence and absence of insulin (Fig. 1b) or its contribution to cardiac ATP production (Fig. 1e). However, insulin did result in a significant increase in cardiac glucose oxidation rates (Fig. 1c) and increased its contribution to cardiac ATP production (Fig. 1e). In contrast, insulin inhibited fatty acid oxidation rates (Fig. 1d) and its contribution to cardiac ATP production (Fig. 1e). Although insulin stimulation enhances glucose contribution to cardiac ATP production at the expense of fatty acid oxidation (Fig. 1c–e), this metabolic shift in cardiac preference for oxidative substrates did not influence cardiac function in the normal heart (Additional file 1: Table S1). There were no significant differences between male and female mice in term of glycolysis, glucose oxidation or fatty acid oxidation (data not shown). Therefore, we combined the data from male and female mice.

**Fig. 2:**
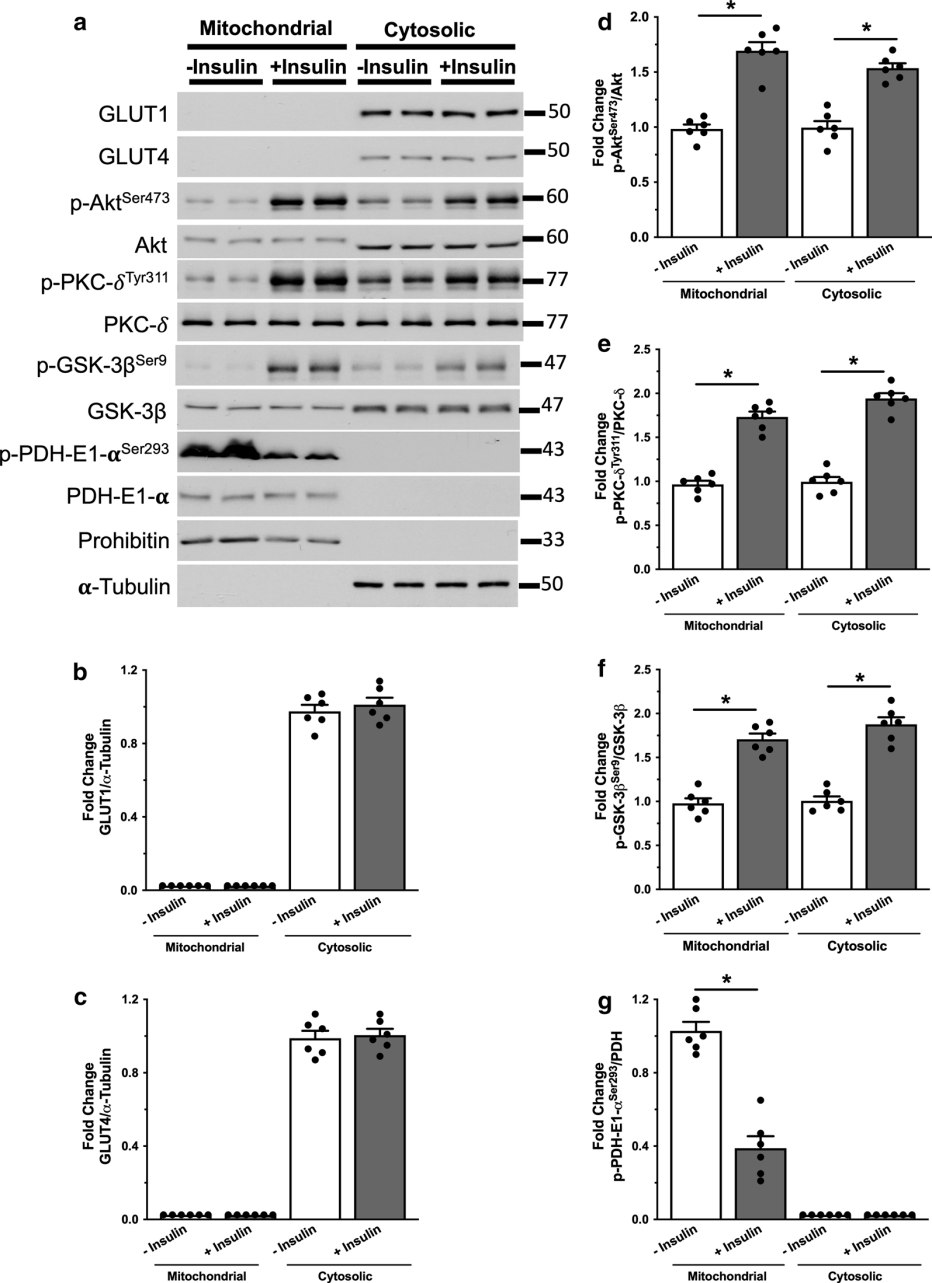
Insulin stimulation of glucose oxidation is associated with enhanced phosphorylation of mitochondrial Akt, PKC-δ and GSK-3β. Hearts were homogenized and fractionated using differential centrifugation to isolate mitochondrial and cytosolic portions. **a** Western blots of GLUT1, GLUT4, Akt, PKC-δ, GSK-3β and PDH and their correspondent phosphorylated serine and tyrosine groups. Prohibitin and α-tubulin as loading control for mitochondrial and cytosolic proteins, respectively. Densitometric analysis of phosphorylated/total levels of **b** GLUT1, **c** GLUT4, **d** Akt, **e** PKC-δ, **f** GSK-3β and **g** PDH in the mitochondria and in the cytosol (n = 6 for each experimental group). Individual values for each group are presented as a scattered plot along with its mean ± SEM. Data were analyzed using Two-way ANOVA followed by Bonferroni correction for multiple comparisons. *p < 0.05 vs—insulin condition in each fraction)

We next examined whether insulin stimulation resulted in an increase in the phosphorylation of mitochondrial Akt, PKC-δ or GSK-3β in the heart. At the end of the perfusion protocol, the heart was homogenized to isolate the cytosolic and mitochondrial fractions. Insulin stimulation did not cause a significant change in the expression of either GLUT1 or GLUT4 in the cytosolic compartment (Fig. 2a–c). However, this direct insulin stimulation of glucose oxidation was accompanied by a significant increase in the phosphorylation of mitochondrial Akt (Akt Ser^473^), PKC-δ (PKC-δ Tyr^311^) and GSK-3β (GSK-3β Ser^9^, Fig. 2a, d–f). Of importance, is that this increase in the phosphorylation of these kinase in the mitochondria was associated with a significant decrease in PDH-E1α Ser^293^ phosphorylation (Fig. 2a, g), indicative of an increase in its activity and consistent with the stimulation of glucose oxidation rates. Collectively, these findings suggest that the direct insulin stimulation of glucose oxidation is associated with enhanced phosphorylation of mitochondrial Akt, PKC-δ and GSK-3β and activation of the PDH complex.

### Inhibition of Akt abolished the direct insulin stimulation of cardiac glucose oxidation rates

**Fig. 3:**
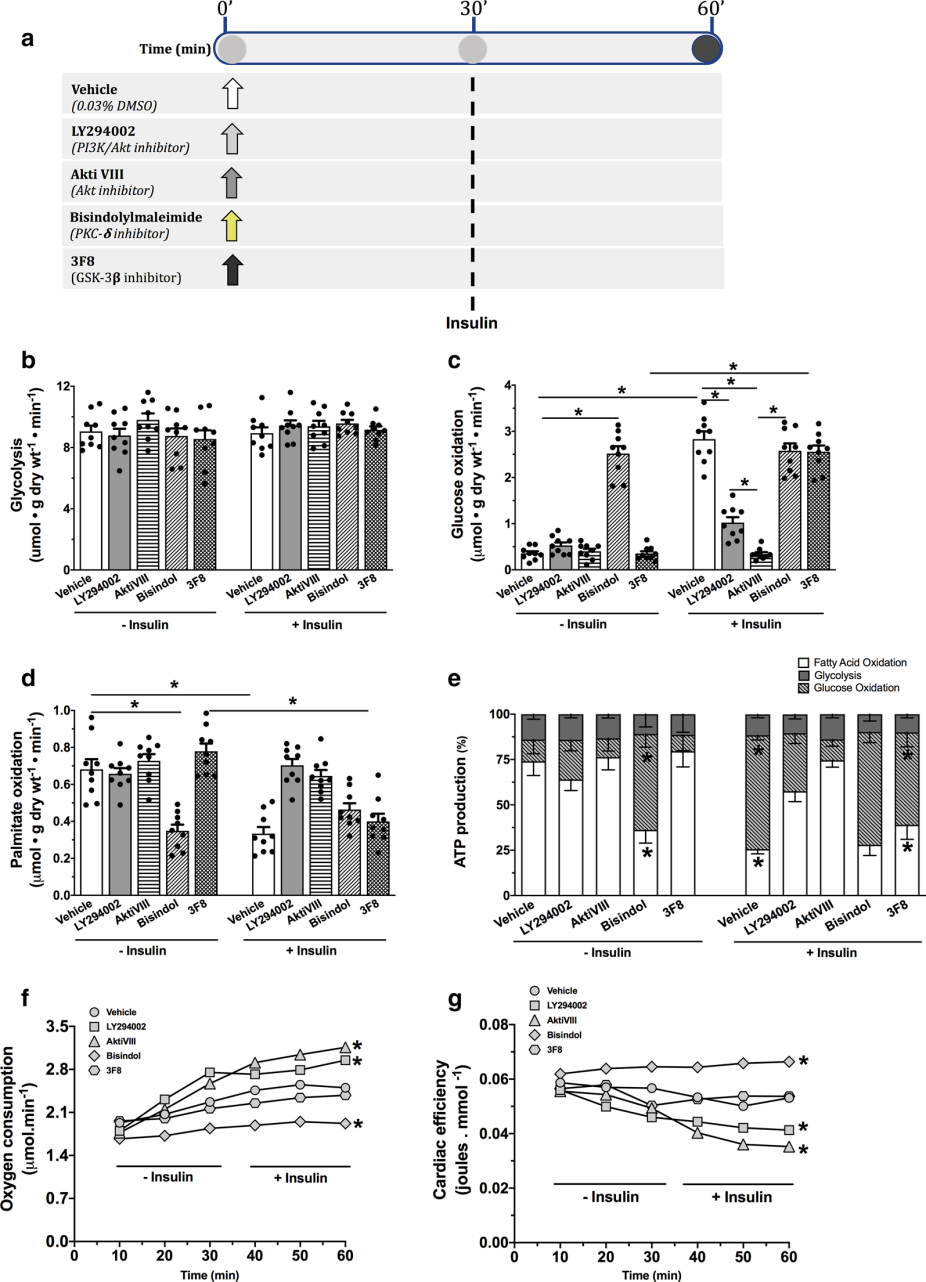
Inhibition of Akt abrogates the direct insulin stimulation of glucose oxidation. **a** Schematic of study design for Study 2. Hearts were perfused in an isolated working heart mode for 30 min following which insulin (100 µU/ml) was added to the perfusate and the hearts were perfused for additional 30 min. The pharmacological modulators of Akt, GSK-3β and PKC-δ were present throughout the perfusion protocol. The metabolic profile of the heart is characterized by measuring **b** glycolysis, **c** glucose oxidation and **d** palmitate (fatty acid) oxidation along with **e** their contribution to cardiac ATP production. **f** Myocardial oxygen consumption and **g** cardiac efficiency (O_2_ consumption/cardiac work) were monitored throughout the perfusion protocol. Arrows indicate the time of adding the vehicle of insulin to the perfusate. Individual values for each group are presented as a scattered plot along with its mean ± SEM (n = 9 for each experimental group). **b**–**e** Were analyzed using Two-way ANOVA followed by Bonferroni correction for multiple comparisons, while **f** and **g** were analyzed using repeated measures ANOVA supported by Bonferroni's post hoc test. For **e**–**g**, *p < 0.05 compared to the vehicle-treated group

We then asked the question whether insulin-stimulated phosphorylation of mitochondrial Akt, PKC-δ and GSK-3β is a prerequisite for transducing insulin stimulation of the mitochondrial PDH complex and glucose oxidation. To determine this, we employed specific pharmacological inhibitors for each of Akt, PKC-δ and GSK-3β to modulate their activities and delineate their involvement in insulin-stimulation of the PDH complex. We randomized isolated working mouse hearts to be perfused in the presence of vehicle or one of the pharmacological inhibitors, namely LY294002 (PI3K/Akt inhibitor), Akti VIII (Akt inhibitor), Bisindolylmaleimide (PKC-δ inhibitor), or 3F8 (GSK-3β inhibitor, Fig. 3a). All hearts were perfused for 30 min in the absence of insulin, following which insulin was then added to the perfusate and the hearts were perfused for an additional 30 min (Fig. 3a). We found that none of the pharmacological inhibitors had any significant effect on cardiodynamics (Additional file 1: Table S2). In addition, none of the pharmacological inhibitors caused any significant change in glycolysis rates or its contribution to the total cardiac ATP production in the presence and absence of insulin compared to the vehicle-treated hearts (Fig. 3b, e). Similar to Study 1, insulin caused a significant increase in cardiac glucose oxidation rates (Fig. 3c, e) and significantly inhibited cardiac fatty acid oxidation rates (Fig. 3d, e) in the vehicle-treated hearts. Of interest, Akt inhibition with either LY294002 or AktiVIII caused a marked abrogation of insulin-stimulated glucose oxidation rates (Fig. 3c, e). Akt inhibition was also accompanied by a significant reduction in the inhibitory effect of insulin on cardiac fatty acid oxidation rates (Fig. 3d, e). This could possibly be a compensatory response to the reduction in glucose oxidation contribution to acetyl CoA for the TCA cycle through the Randle cycle [26]. This increase reliance on fatty acid oxidation at the expense of glucose oxidation was accompanied by a significant increase in cardiac oxygen consumption rates by ~ 20–26% (Fig. 3f), since fatty acid is less oxygen efficient oxidative substrate compared to glucose [27, 28]. As a result, Akt inhibition resulted in a significant decrease in cardiac efficiency (cardiac work/O_2_ consumed) by 22–35% compared to vehicle-treated hearts (Fig. 3g).

We also found that inhibition of PKC-δ using Bisindolylmaleimide significantly increased glucose oxidation rates in hearts perfused in the absence of insulin (Fig. 3c, e). Interestingly, there was no further increase in glucose oxidation rates following insulin stimulation (Fig. 3c, e), suggesting that PKC-δ inhibition mimics the direct insulin stimulation of glucose oxidation. In support of these findings, inhibition of PKC-δ significantly limited fatty acid oxidation rates in the heart in the absence of insulin, with no further inhibitory effect in the presence of insulin (Fig. 3d, e). Inhibition of PKC-δ also caused a significant decrease in cardiac oxygen consumption by ~ 25% (Fig. 3f) and improved cardiac efficiency by ~ 25% (Fig. 3g).

We also found that inhibition of GSK-3β with 3F8 did not have a significant effect on either the basal or insulin-stimulated glucose oxidation rates (Fig. 3c, e). Furthermore, GSK-3β inhibition did not cause a significant change in cardiac fatty acid oxidation rates (Fig. 3d, e). Consistent with that, we also did not observe any significant effect of GSK-3β inhibition on either cardiac oxygen consumption rates or cardiac efficiency (Fig. 3f, g). Unlike Akt and PKC-δ, these findings suggest that mitochondrial translocation of GSK-3β following insulin stimulation does not play an important role in mediating the direct insulin stimulation of cardiac glucose oxidation.

### Activation of mitochondrial Akt is a prerequisite to mediate direct insulin stimulation of glucose oxidation

**Fig. 4:**
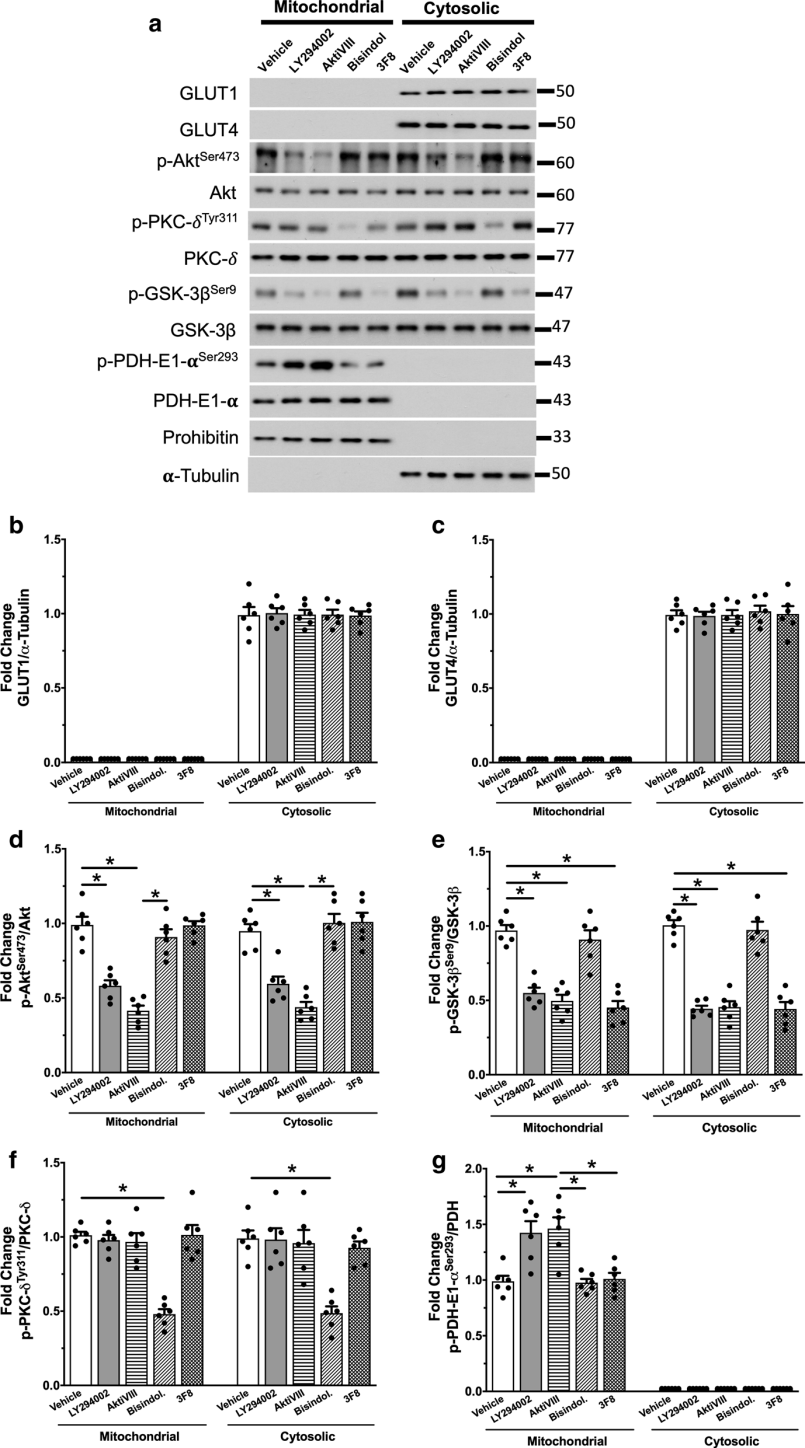
Inhibition of mitochondrial Akt abrogates insulin stimulation of PDH. Hearts from the second series of perfusions with the pharmacological inhibitors (as shown in Fig. 3) were homogenized and fractionated using differential centrifugation to isolate mitochondrial and cytosolic portions. **a** Western blots of Western blots of GLUT1, GLUT4, Akt, PKC-δ, GSK-3β and PDH and their correspondent phosphorylated serine and tyrosine groups. Prohibitin and α-tubulin were employed as loading controls for mitochondrial and cytosolic proteins, respectively. Densitometric analysis of phosphorylated/total levels of **b** GLUT1, **c** GLUT4, **d** Akt, **e** GSK-3β, **f** PKC-δ and **g** PDH in the mitochondria and in the cytosol. Individual values for each group are presented as a scattered plot along with its mean ± SEM (n = 6 for each experimental group). Data were analyzed using Two-way ANOVA followed by Bonferroni post hoc test for multiple comparisons (*p < 0.05)

We next asked the question whether the observed alterations in mitochondrial glucose and fatty acid oxidation rates with the pharmacological inhibitors are due to modulating the activity of mitochondrial of Akt, PKC-δ or GSK-3β. We found that none of the pharmacological inhibitors had any significant effect on either GLUT1 or GLUT4 expression (Fig. 4a–c). We also found that the inhibition of insulin stimulation of glucose oxidation with Akt inhibitors was associated with abrogation of the phosphorylation of mitochondrial Akt Ser^473^ (Fig. 4d). This abrogation of mitochondrial Akt was also accompanied by inhibition of the insulin-induced activation of the PDH complex (Fig. 4g), confirming that insulin-induced phosphorylation of mitochondrial Akt is a prerequisite in mediating the direct insulin stimulation of the PDH complex and cardiac glucose oxidation.

There have been some suggestions that the activity of PDH complex can be regulated by PKC-δ [19–21, 29]. However, whether PKC-δ activates or inhibits the PDH complex activity is still under debate. Our study revealed a mechanism through which PKC-δ modulates PDH complex activity. We found that the stimulatory effect of PKC-δ inhibition on the rates of glucose oxidation in the absence of insulin was associated with significant inhibition of mitochondrial PKC-δ Tyr^311^ (Fig. 4e). Inhibition of mitochondrial PKC-δ was associated with a significant activation of the PDH complex (Fig. 4g), which is consistent with enhanced cardiac glucose oxidation rates in the absence of insulin. This suggests an inhibitory effect of mitochondrial PKC-δ on the PDH complex and glucose oxidation. Interestingly, the presence of insulin in the Bisindolylmaleimide-treated hearts did not cause a further increase in PDH complex activity (Fig. 4g). This is consistent with the absence of a further stimulation of glucose oxidation by insulin following PKC-δ inhibition, suggesting a common mechanism through which mitochondrial PKC-δ and insulin can modulate the PDH complex.

It has also been proposed that PKC-δ can influence the activity of Akt along with its downstream effectors [21, 30–32]. In our study, we found that inhibition of mitochondrial PKC-δ significantly enhanced mitochondrial Akt activity (Fig. 4d). Interestingly, the presence of insulin did not cause any further enhancement of mitochondrial Akt activity (Fig. 4d). Therefore, it seems plausible that mitochondrial PKC-δ play a critical role as a negative feedback loop to limit cardiac glucose oxidation via inhibiting mitochondrial Akt and, as a result, limits the PDH complex activity. These findings further emphasize that mitochondrial Akt is prerequisite for mediating the direct insulin stimulation of glucose oxidation. It also suggests that inhibition of PKC-δ can be an alternative approach to stimulate PDH complex in the heart in an insulin-independent manner, although this hypothesis needs to be directly investigated in future studies.

We also found that the phosphorylation of mitochondrial GSK-3β Ser^9^ was significantly inhibited by 3F8 as well as by Akt inhibitors (Fig. 4a, f). However, inhibition of the phosphorylation of mitochondrial GSK-3β did not influence insulin-stimulation of the PDH complex (Fig. 4a, g). In line with the absence of any significant effect of mitochondrial GSK-3β inhibition on the direct insulin stimulation of glucose oxidation, these findings confirm that mitochondrial GSK-3β does not mediate the direct insulin stimulation of glucose oxidation.

## Discussion

This study revealed, for the first time, a number of novel and important findings. First, insulin stimulation of PDH complex and cardiac glucose oxidation rates is associated with rapid and robust phosphorylation of mitochondrial Akt, GSK-3β and PKC-δ. Second, mitochondrial Akt is essential for the direct stimulation of insulin to cardiac glucose oxidation, independent of glucose uptake and glycolysis. Third, inhibition of mitochondrial Akt increases myocardial oxygen consumption and compromises cardiac efficiency. Fourth, inhibition of mitochondrial PKC-δ mimics the direct insulin stimulation of cardiac glucose oxidation. Fifth, inhibition of mitochondrial PKC-δ enhances the activity of mitochondrial Akt and improves cardiac efficiency. Sixth, mitochondrial GSK-3β is not implicated in the direct stimulation of insulin on cardiac glucose oxidation.

**Fig. 5:**
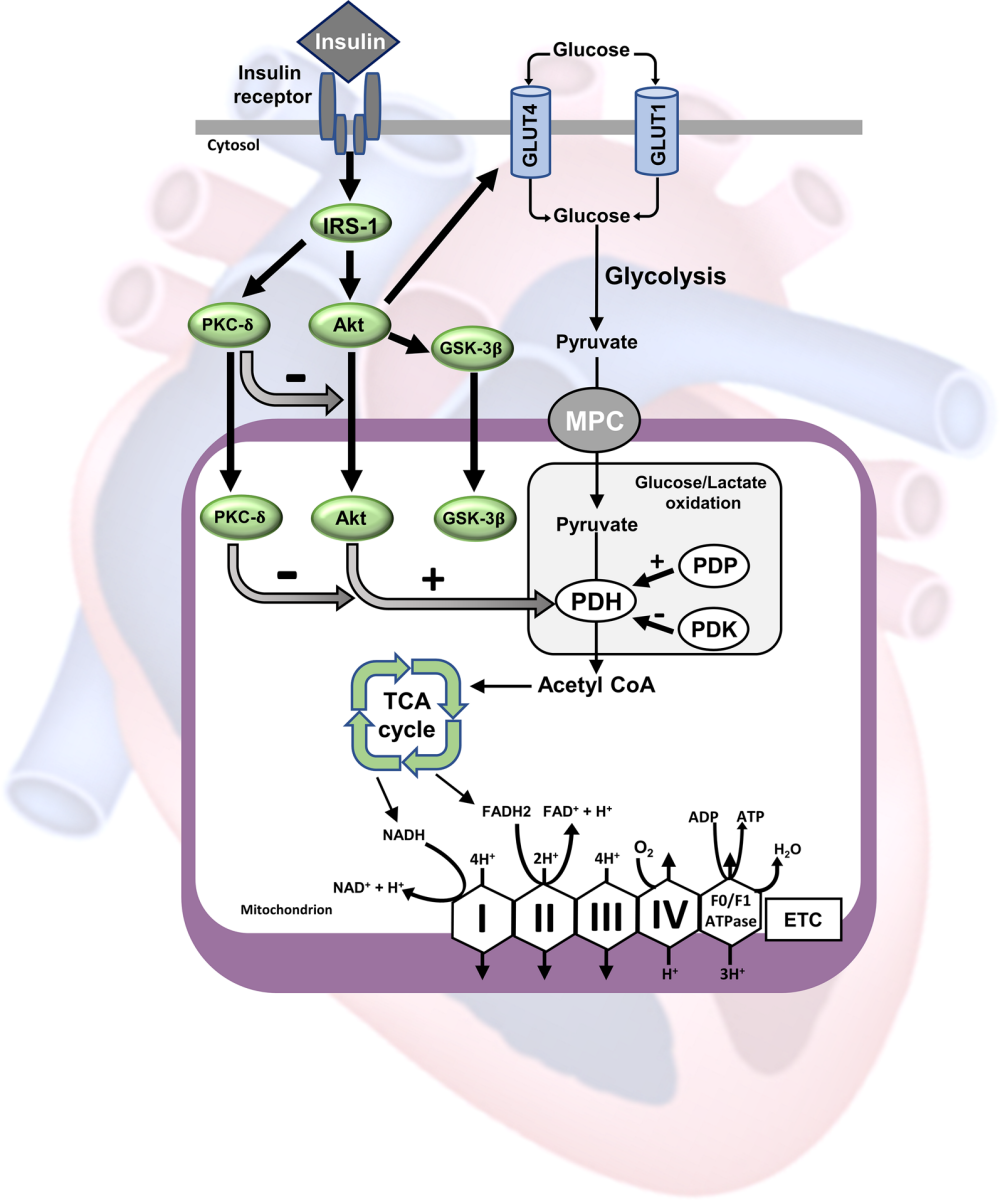
Schematic summary of the direct stimulation of insulin to glucose oxidation in the heart. Insulin stimulates mitochondrial protein kinase B (Akt), protein kinase C-delta (PKC-δ) and glycogen synthase kinase-3 beta (GSK-3β). Mitochondrial Akt plays a prerequisite role in mediating the activation of pyruvate dehydrogenase (PDH) and stimulation of cardiac glucose oxidation. Mitochondrial PKC-δ acts as a negative feedback loop by opposing insulin action in the heart and limiting the activity of mitochondrial Akt. Mitochondrial GSK-3β is not involved in mediating the direct stimulation of insulin on cardiac glucose oxidation. *PDP* pyruvate dehydrogenase phosphatase, *PDK* pyruvate dehydrogenase kinase, *MPC* mitochondrial pyruvate carrier, *GLUT1* insulin-independent glucose transporter, *GLUT4* insulin-dependent glucose transporter

While the interplay between glucose oxidation and fatty acid oxidation and their contribution to cardiac ATP production can largely be explained by the phenomena that is called the Randle cycle [26], insulin plays a critical role in regulating the heart’s preference for these oxidative substrates. Insulin inhibits cardiac fatty acid oxidation via abrogating the inhibitory effect of 5′AMP-activated protein kinase (AMPK) on acetyl CoA carboxylase (ACC) and increasing malonyl CoA, a potent inhibitory of mitochondrial fatty acid uptake [33, 34]. Moreover, insulin stimulates glucose uptake that increases glycolytic rates and subsequently mitochondrial glucose oxidation rates. It has also been shown that insulin can directly stimulate glucose oxidation independent of enhancing glucose uptake or glycolysis [11–14]. The hallmark of this direct stimulation of glucose oxidation by insulin is the dephosphorylation and activation of mitochondrial PDH complex [11–14]. However, the exact mechanism through which insulin signal is transmitted form the cell membrane to the mitochondria to activate PDH complex has not been previously elucidated. Here, we show that this direct insulin stimulation of the PDH complex is associated with increased phosphorylation of mitochondrial Akt, GSK-3β and PKC-δ. We then asked the question whether any of these kinases plays an indispensable role in transducing insulin signal to the PDH complex. Therefore, we investigated how inhibiting the phosphorylation of each one of these kinases in the mitochondrial will influence insulin-stimulated PDH complex using pharmacological modulators for these kinases. Akt is an important component of the insulin signaling pathway and it mediates the majority of the metabolic actions of insulin. It enhances glucose uptake by triggering the translocation of insulin-dependent glucose transporter-4 (GLUT4) to the cell membrane. It also regulates the activity of another downstream effector, namely GSK-3β, through increasing its phosphorylation at its serine 9 residue and inhibiting its activity. Impaired activity of Akt is positively correlated with reduced glucose oxidation [3, 4]. It has also been shown that Akt can be translocated the nucleus to regulate the activity of GSK-3β during the cell cycle [35]. It has also been shown that Akt can be translocated to the mitochondrial following insulin stimulation, an effect which is associated with modulating mitochondrial bioenergetics [17, 36]. However, how mitochondrial translocation of Akt influences mitochondrial oxidative phosphorylation is not known. In this study, we found that insulin-stimulated PDH complex is associated with enhanced phosphorylation of mitochondrial Akt. We also found that inhibition of mitochondrial Akt with either LY294002 or AktiVIII abolishes the direct stimulatory effect of insulin on PDH complex and cardiac glucose oxidation rates. This confirms a prerequisite role of mitochondrial Akt in mediating the direct stimulation of insulin on the PDH complex (Fig. 5). Of importance, is that pharmacological inhibition of mitochondrial Akt was not accompanied by a significant change in glycolysis rates. This is important since it confirms that the observed inhibition of glucose oxidation following the inhibition of mitochondrial Akt was not secondary to the reduction in either glucose uptake or glycolysis.

The decrease in glucose oxidation rates following Akt inhibition with either of the pharmacological inhibitors was accompanied by an expected increase in fatty acid oxidation (based on the Randle cycle). Indeed, we directly measured fatty acid oxidation rates in these hearts and we found that fatty acid oxidation rates were increased from (0.4 μmol/g dry wt/min to 0.8 μmol/g dry wt/min (resulting in a ~ 3000–4000 μmol/g dry wt/min increase in acetyl CoA production from fatty acid oxidation), which matched the reduction acetyl-CoA production from glucose oxidation rates (~ 3000–4000 μmol/g dry wt/min reduction) following Akt inhibition (Fig. 3c, d). We also directly measured myocardial oxygen consumption rates in these hearts, and found that the increase in fatty acid oxidation following Akt inhibition was accompanied by a significant increase in myocardial oxygen consumption rates (Fig. 3f). As a result, the increase in fatty acid oxidation was accompanied by a significant decrease in cardiac efficiency in these hearts (Fig. 3g), probably due to the fact that fatty acid is a less oxygen-efficient substrate compared to glucose [27, 28]. A mismatch between cardiac fatty acid supply and utilization has been link to the accumulation of ceramide and diacylglycerol (DAG) along with reactive oxygen species (ROS) generation [37]. However, it is unlikely that this increase in fatty acid oxidation rates lead to the accumulation of ceramide and DAG. In fact, this increase in fatty acid oxidation rates following Akt inhibition, in the hearts perfused in the presence of the same fatty acid concentrations as control hearts, would likely facilitate the removal of cytoplasmic fatty acid moieties that may be destined for ceramide or DAG synthesis in the myocardium.

A decrease in glucose oxidation rates has the potential to feedback and modulate glycolysis, hexosamine biosynthesis, polyols biosynthesis, the pentose phosphate pathway, and the advanced glycation end products pathway). In our study, we directly measured glycolysis rates by measuring the amount of ^3^H_2_O released from 5-[^3^H]glucose at the triosephosphate isomerase and enolase steps of glycolysis, enzymes near the end of the glycolytic pathway [38]. We found that insulin stimulation did not cause a significant change in cardiac glycolysis rates (Fig. 1b). In addition, none of the pharmacological inhibitors caused any significant change in cardiac glycolysis rates (Fig. 3b). Since glucose flux through all of the hexosamine, polyols, pentoses, and AGEs pathways occurs before glucose enters the enolase step of glycolysis, our data suggest that inhibition of glucose oxidation with LY294002 or AktiVIII were not likely to effect on glucose flux through any of these pathways since glucose flux through enolase was not changed. It also needs to be emphasized that changes in cardiac glucose oxidation rates, at least in the mouse heart, are more likely to modify the fate of pyruvate, whether it would be reduced to lactate or oxidized to acetyl CoA, and less likely to influence glucose uptake and glycolysis. In support of that, we showed that insulin can directly stimulate glucose oxidation with no significant effect on glycolysis. In addition, we also showed that Akt inhibition with either LY294002 or AktiVIII significantly inhibited glucose oxidation (Fig. 3c) with no significant effect on glycolysis rates.

It has been suggested that PKC-δ modulates the activity of PDH complex through an unknown mechanism [19–21, 29]. However, data have been previously inconclusive in this context. For instance, insulin stimulation of the PDH complex was blocked by PKC-δ inhibition in Zajdela hepatoma cultured cells [29]. In contrast, insulin-stimulated PKC-δ is shown to inhibit the PDH complex in fibroblasts [20]. Furthermore, activation of PKC-δ at reperfusion is associated with deactivation of PDH complex and impaired post-ischemic recovery [21]. In this study, we found that inhibition of mitochondrial PKC-δ stimulates PDH complex and cardiac glucose oxidation, in a glycolysis-independent manner. Moreover, inhibition of mitochondrial PKC-δ results in stimulation of mitochondrial Akt that is comparable to insulin’s effect. A number of studies have suggested an inhibitory effect of PKC-δ on Akt signaling. For example, it has been shown that PKC-δ activation in the reperfused rat heart is associated with decreased Akt activity and increased GSK activity [21, 30–32]. PKC-δ-induced inhibition of Akt has also been linked to a decrease in the activity of the PDH complex at reperfusion [21]. In this study, we showed that insulin stimulates the phosphorylation of mitochondrial Akt, and that this is associated with increased activity of the PDH complex. We also showed that PKC-δ inhibition can mimic the effect of insulin by enhancing the activity of mitochondrial Akt, activation of PDH complex and stimulation of cardiac glucose oxidation. Taken together, we propose that PKC-δ has an inhibitory effect on mitochondrial Akt and inhibition of PKC-δ stimulates cardiac glucose oxidation via enhancing Akt activity in the mitochondria. These findings also suggest that mitochondrial PKC-δ plays a critical role in regulating the activity of mitochondrial Akt and the PDH complex. It also suggests that inhibition of PKC-δ can be an alternative approach to stimulate PDH complex in the heart in an insulin-independent manner (Fig. 5). However, this hypothesis needs to be directly investigated in future studies.

There are a number of caveats for this study. It is not clear how Akt, PKC-δ or GSK-3β crosses the mitochondrial membrane and whether they enter the mitochondria via a specific channel/pore or through passive diffusion. Moreover, it is still not clear what the exact function of mitochondrial GSK-3β is. Furthermore, the mechanism through which mitochondrial Akt trigger the activity of the PDH complex is yet to be determine. Furthermore, the pharmacological inhibitors of Akt, PKC-δ, GSK-3β were very effective in inhibiting the activities of these kinases (Fig. 4). However, whether these inhibitors can also modulate the activity of other isoforms of these kinases is not clear. While insulin stimulation caused a significant increase in the levels of phosphorylated Akt, PKC-δ and GSK-3β in the mitochondrial, this was not accompanied by a significant change in the total levels of these kinases in the mitochondria. This may suggest that these kinases can be phosphorylated inside the mitochondria. However, there is no well-defined mechanism through which these kinases can be phosphorylated in the mitochondria. This may also suggest that these kinases are present in the mitochondria and that insulin stimulates their mitochondrial translocation of the phosphorylated forms of theses kinases in relatively small levels, compared to the total levels of the kinases. However, this hypothesis needs to be directly investigated. It is also important to note that the mechanism(s) through which these kinases may enter into the mitochondria following insulin stimulation is not known. Moreover, it is also not clear how these kinases move back (i.e. “recycled”) from the mitochondria to the cytosol or the dynamics of this movement. It is also not known whether these kinases can be dephosphorylated in the mitochondria or they can only be dephosphorylated in the cytosol. It is important to highlight that previous studies have employed transgenic mouse lines to investigate the impact chronic activation of Akt on cardiac function and structure. For instance, it has been shown that chronic overexpression of Akt leads to dilated cardiomyopathy [39] and cardiac dysfunction [40–43]. However, conditional transgenic overexpression of Akt preserves cardiac function [39, 43]. Unfortunately, we could not find any study that investigated the impact of Akt overexpression on the direct insulin stimulation of glucose oxidation. All of these questions represent very interesting areas for future investigations.

## Conclusions

In conclusion, this study is the first to show that phosphorylation of mitochondrial Akt is a prerequisite for the direct insulin stimulation of cardiac glucose oxidation. This study also provides novel mechanistic insights into the critical role of mitochondrial PKC-δ in regulating the PDH complex activity via modulating mitochondrial Akt activity and that PKC-δ inhibition mimics insulin stimulation of mitochondrial Akt and glucose oxidation. This suggests that targeting mitochondrial Akt is a potential therapeutic approach to improve cardiac insulin-stimulated glucose oxidation in the setting of heart failure, obesity and diabetes, situations where the heart is insulin resistant.

## Supplementary information

** Additional file 1: Table S1.** Cardiodynamics of hearts perfused in the absence or presence of insulin. **Table S2.** The impact of the pharmacological inhibitors on cardiodynamics in hearts perfused the absence or presence of insulin.

## Data Availability

The datasets used and/or analyzed during the current study are available from the corresponding authors on reasonable request.

## Abbreviations

ACC: Acetyl CoA carboxylase
Akt: Protein kinase A
AMPK: 5ʹ AMP-activated protein kinase
GLUT1: Glucose transporter type 1
GLUT4: Glucose transporter type 4
GSK-3β: Glycogen synthase kinase-3 beta
IRS-1: Insulin receptor substrate 1
MPC: Mitochondrial pyruvate carrier
PI3K: Phosphoinositide 3-kinase
PKC-δ: Protein kinase C-delta
PDH: Pyruvate dehydrogenase
TCA: Tricarboxylic acid
