# Supplementary material for: Insulin directly stimulates mitochondrial glucose oxidation in the heart
Source: Cardiovasc Diabetol. 2020 Dec 7;19:207. doi: 10.1186/s12933-020-01177-3 (PMC7722314; doi:10.1186/s12933-020-01177-3)
Supplement: Supplementary file 1 — Additional file 1: Table S1. Cardiodynamics of hearts perfused in the absence or presence of insulin. Table S2. The impact of the pharmacological inhibitors on cardiodynamics in hearts perfused the absence or presence of insulin. [file 12933_2020_1177_MOESM1_ESM.docx]

**Additional file**

**Tables**

**Table S1:** Cardiodynamics of hearts perfused in the absence or presence of insulin

| **Parameter** | **Without insulin**  **(n=9)** | **With Insulin**  **(n=9)** |
| --- | --- | --- |
| **Heart rate (beat per minute)** | 315 ± 7 | 319 ± 8 |
| **Cardiac Output (ml.min^-1^)** | 12.1 ± 0.8 | 12.3 ± 0.6 |
| **Aortic Flow (ml.min^-1^)** | 10.7 ± 0.5 | 10.2 ± 0.5 |
| **Coronary Flow (ml.min^-1^)** | 2.76 ± 0.14 | 2.81 ± 0.18 |
| **Cardiac Work (joules.min^-1^)** | 0.078 ± 0.005 | 0.077 ± 0.006 |

**Table S2:** The impact of the pharmacological inhibitors on cardiodynamics in hearts perfused the absence or presence of insulin

| **Parameter** | **Control**  **(n=9)** | **LY294022**  **(n=9)** | **AktiVIII**  **(n=9)** | **Bisindolylmaleimide**  **(n=9)** | **3F8**  **(n=9)** |
| --- | --- | --- | --- | --- | --- |
| **Heart rate (beat per minute)** | 330 ± 7 | 326 ± 8 | 320 ± 8 | 319 ± 8 | 320 ± 8 |
| **Cardiac Output (ml.min^-1^)** | 13.0 ± 0.8 | 12.2 ± 0.6 | 12.3 ± 0.8 | 12.3 ± 0.8 | 12.9 ± 0.7 |
| **Aortic Flow (ml.min^-1^)** | 10.3 ± 0.5 | 10.2 ± 0.5 | 10.4 ± 0.6 | 11.6 ± 0.5 | 10.5 ± 0.6 |
| **Coronary Flow (ml.min^-1^)** | 2.60 ± 0.15 | 2.59 ± 0.20 | 2.57 ± 0.17 | 2.79 ± 0.16 | 2.88 ± 0.14 |
| **Cardiac Work (joules.min^-1^)** | 0.098 ± 0.009 | 0.083 ± 0.006 | 0.085 ± 0.007 | 0.089 ± 0.01 | 0.083 ± 0.01 |
